# Fungal Metabolomics and Genomics: Integrating Multi-Omics to Decipher Function, Diversity, and Application

**DOI:** 10.3390/jof12050365

**Published:** 2026-05-15

**Authors:** Chengwei Liu, Jianzhao Qi, Xiuzhang Li

**Affiliations:** 1 College of Life Science, Northeast Forestry University, Harbin 150040, China; 2 Shaanxi Key Laboratory of Natural Products & Chemical Biology, College of Chemistry & Pharmacy, Northwest A&F University, Xianyang 712100, China; 3 State Key Laboratory of Plateau Ecology and Agriculture, Qinghai Academy of Animal and Veterinary Sciences, Qinghai University, Xining 810016, China

## 1. Introduction

Fungi represent one of the most diverse groups of organisms on Earth, playing fundamental roles in ecosystems, agriculture, and biotechnology. Their remarkable metabolic versatility is closely linked to complex genomic architectures and highly regulated biosynthetic systems. With the rapid development of high-throughput sequencing, genomics, metabolomics, proteomics, and other multi-omics technologies, fungal research has entered a new era in which genotype–phenotype–metabolite relationships can be systematically explored. Fungal metabolomics and genomics provide powerful complementary perspectives: genomics reveals the genetic potential of fungi, particularly biosynthetic gene clusters and adaptive evolution, while metabolomics reflects the functional output of these genetic programs under specific environmental and developmental conditions. The integration of these approaches has greatly accelerated the discovery of novel natural products, the understanding of fungal development, and the elucidation of ecological interactions.

These topics are addressed in the Special Issue “Fungal Metabolomics and Genomics”, which comprises 19 original research articles covering genome sequencing and comparative analysis, multi-omics integration of metabolic regulation, and functional studies of fungal secondary metabolism and ecological interactions. The contributions collectively highlight the expanding role of fungi in biotechnology, agriculture, medicine, and environmental adaptation, and are briefly summarized in the following sections.

## 2. Overview of Published Articles

A significant portion of the Special Issue focuses on fungal genome sequencing, comparative genomics, and evolutionary analysis. Several studies provide high-quality genome assemblies that improve taxonomic resolution and reveal evolutionary adaptation. Qi et al. (contribution 1) reported a haplotype-phased chromosome-level genome assembly of *Floccularia luteovirens*, uncovering its adaptive evolution and biosynthetic potential. Bai et al. (contribution 2) conducted comparative genomic analysis of *Inonotus hispidus* across different host trees, revealing host-associated genomic variation. He et al. (contribution 3) presented genome sequencing and fine mapping of *Pholiota nameko*, providing valuable genetic resources for future breeding. Wang et al. (contribution 4) analyzed genomic differences between two monokaryons of *Auricularia heimuer*, shedding light on domestication processes. Cheng et al. (contribution 5) investigated genomic determinants of cobweb disease resistance in *Agaricus bisporus*, while Yang et al. (contribution 6) provided genome-based identification of *Alternaria* species. Sigova et al. (contribution 7) generated telomere-to-telomere genome assemblies of *Colletotrichum lini*, greatly improving structural genome resolution in pathogenic fungi. Collectively, these studies expand fungal genomic resources and deepen our understanding of fungal evolution and adaptation.

Another major theme concerns integrative multi-omics analyses of fungal metabolism, development, and stress responses. Lu et al. (contribution 8) investigated *Auricularia heimuer* under high-temperature stress using integrated transcriptomics and metabolomics, revealing coordinated regulatory responses. Huo et al. (contribution 9) studied degeneration mechanisms in *Morchella importuna* during repeated subculturing, identifying metabolic and transcriptional reprogramming. Tang et al. (contribution 10) integrated proteomics and metabolomics to compare wild and cultivated *Ophiocordyceps sinensis*, revealing quality-associated metabolic differences. A related study by Tang et al. (contribution 11) on *Cordyceps* species further highlighted amino acid metabolism and protein quality divergence. Wang et al. (contribution 12) and Liu et al. (contribution 13) combined transcriptomics and metabolomics to explore terpenoid biosynthesis in *Amylostereum areolatum* and fruiting body formation in *Sarcomyxa edulis*, respectively. These studies collectively demonstrate how multi-omics integration enables a deeper understanding of fungal development and metabolic regulation.

A third group of studies focuses on fungal secondary metabolism, ecological functions, and biotechnological applications. Ma et al. (contribution 14) explored the metabolomic and genomic basis of bioactive compounds in *Aspergillus terreus*, highlighting its potential anti-Alzheimer’s disease activity. Xiao et al. (contribution 15) investigated how different drying methods influence metabolite profiles and antioxidant activity in *Floccularia luteovirens*. Shabaev et al. (contribution 16) integrated secretome, metabolome, and genomic analyses of *Crucibulum laeve*, revealing lignocellulose degradation systems and metabolic pathways. Waheed et al. (contribution 17) characterized xylanase gene families in *Thermothelomyces fergusii*, providing insights into industrial enzyme potential. Villanueva et al. (contribution 18) analyzed genomes and effector proteins of *Phytopythium vexans*, elucidating mechanisms of plant pathogenicity. Wu et al. (contribution 19) resolved the 3D genome structure of *Exophiala tremulae* and demonstrated its role in plant–fungus symbiosis and growth promotion. Liu et al. (contribution 13) investigated transcriptomic regulation of *Sarcomyxa edulis* fruiting body formation, linking developmental biology with environmental adaptation. These studies highlight the ecological diversity and biotechnological potential of fungi across multiple systems.

## 3. Conclusions

Overall, the 19 contributions in this Special Issue illustrate the rapid progress of fungal metabolomics and genomics as an integrated research field. A clear trend emerges toward combining genome sequencing with multi-omics approaches to link genetic potential with metabolic output and ecological function. This integration not only enhances our understanding of fungal evolution and development but also facilitates the discovery of novel bioactive compounds and industrially relevant enzymes. The studies presented here collectively demonstrate that fungi are not only key ecological players but also valuable resources for biotechnology, agriculture, and medicine. We anticipate that continued advances in sequencing technologies, metabolomics platforms, and computational biology will further accelerate discoveries in this field and deepen our understanding of fungal systems.

As Guest Editors, we sincerely thank all authors and reviewers for their valuable contributions, and we acknowledge the support of the editorial team of the *Journal of Fungi.* We hope this Special Issue will serve as a useful reference for researchers and stimulate further progress in fungal metabolomics and genomics.

